# Alterations in Peripheral Lymphocyte Subsets under Immunochemotherapy in Stage IV SCLC Patients: Th17 Cells as Potential Early Predictive Biomarker for Response

**DOI:** 10.3390/ijms25105056

**Published:** 2024-05-07

**Authors:** Ann-Kristin Schmälter, Phillip Löhr, Maik Konrad, Johanna Waidhauser, Tim Tobias Arndt, Stefan Schiele, Alicia Thoma, Björn Hackanson, Andreas Rank

**Affiliations:** 1 Department of Hematology and Oncology, Augsburg University Hospital and Medical Faculty, Comprehensive Cancer Center Augsburg, 86156 Augsburg, Germany; phillip.loehr@uk-augsburg.de (P.L.); maik.konrad@uk-augsburg.de (M.K.); johanna.waidhauser@uk-augsburg.de (J.W.); alicia.thoma@uk-augsburg.de (A.T.); bjoern.hackanson@uk-augsburg.de (B.H.); andreas.rank@uk-augsburg.de (A.R.); 2 Bavarian Cancer Research Center (BZKF), 86156 Augsburg, Germany; 3 Institute of Mathematics, University of Augsburg, 86159 Augsburg, Germany; tobias.arndt@uka-science.de (T.T.A.); stefan.schiele@math.uni-augsburg.de (S.S.); 4 Department of Medicine I, Medical Center, Faculty of Medicine, University of Freiburg, 79106 Freiburg, Germany

**Keywords:** small-cell lung cancer, immunochemotherapy, Th 17 cells, predictive biomarker, cellular immune status

## Abstract

UICC stage IV small-cell lung cancer (SCLC) is a highly aggressive malignancy without curative treatment options. Several randomized trials have demonstrated improved survival rates through the addition of checkpoint inhibitors to first-line platin-based chemotherapy. Consequently, a combination of chemo- and immunotherapy has become standard palliative treatment. However, no reliable predictive biomarkers for treatment response exist. Neither PD-L1 expression nor tumor mutational burden have proven to be effective predictive biomarkers. In this study, we compared the cellular immune statuses of SCLC patients to a healthy control cohort and investigated changes in peripheral blood B, T, and NK lymphocytes, as well as several of their respective subsets, during treatment with immunochemotherapy (ICT) using flow cytometry. Our findings revealed a significant decrease in B cells, while T cells showed a trend to increase throughout ICT. Notably, high levels of exhausted CD4+ and CD8+ cells, alongside NK subsets, increased significantly during treatment. Furthermore, we correlated decreases/increases in subsets after two cycles of ICT with survival. Specifically, a decrease in Th17 cells indicated a better overall survival. Based on these findings, we suggest conducting further investigation into Th17 cells as a potential early predictive biomarkers for response in patients receiving palliative ICT for stage IV SCLC.

## 1. Introduction

Small-cell lung cancer (SCLC) is an aggressive malignancy, and diagnosis is often made in advanced-stage disease. For UICC stage IV SCLC, no curative treatment options exist. Several randomized trials (e.g., IMpower 133 and CASPIAN) have shown that the addition of a checkpoint inhibitor (CPI) like atezolizumab or Durvalumab to first-line platin-based chemotherapy improves overall survival by roughly two months. Therefore, a combination of immunochemotherapy (ICT) is standard palliative treatment. Median overall survival (OS) is approximately 12 to 13 months with ICT [1,2]. 

Thus far, no robust biomarkers exist to predict whether a patient with SCLC responds to ICT [3,4,5]. In non-small-cell lung cancer (NSCLC), programmed cell death ligand-1 (PD-L1) status is used to guide treatment decisions as a high PD-L1 score correlates with good response to immune checkpoint inhibition [6]. Another prognostic factor is tumor mutational burden (TMB). High TMB is associated with poor prognosis [7]. SCLC cells, however, express PD-L1 only rarely. Neither the IMpower 133 nor the CASPIAN trial could show that PD-L1 expression or TMB were predictive for the efficacy of atezolizumab or Durvalumab, respectively [1,2]. Based only on these markers, no statement can be made about the prognoses of individual patients. 

In this pilot study, we focused on analyzing the cellular immune composition in stage IV SCLC patients receiving palliative ICT with carboplatin, etoposide and atezolizumab, a humanized, monoclonal antibody against PD-L1. The cellular immune system has an important role as an effector of the anti-tumor immune response triggered by CPI. We compared lymphocyte subsets in peripheral blood from SCLC patients with healthy controls, considering age and gender to detect abnormalities. Further, patients’ lymphocyte statuses were monitored longitudinally during ICT and CPI maintenance therapy. We investigated the influence of an increase or decrease in each peripheral blood lymphocyte subset after two cycles of ICT on survival to identify potential biomarkers in the cellular immune system that predict early response to ICT treatment. Such a marker could help to stop or change treatment to avoid side effects of ICT. 

## 2. Results

### 2.1. Patient Characteristics

The median age of patients was 70 years (range 44–83), and 41% were female (see Supplemental Table S3 for details). Median follow-up was 28 months. At last follow-up, 9 out of 32 patients were still alive, 20 had died within one year after diagnosis, and 13 had died within 6 months after diagnosis. 

### 2.2. Comparison to Healthy Control Group via Multivariable Analysis

**Figure 1:**
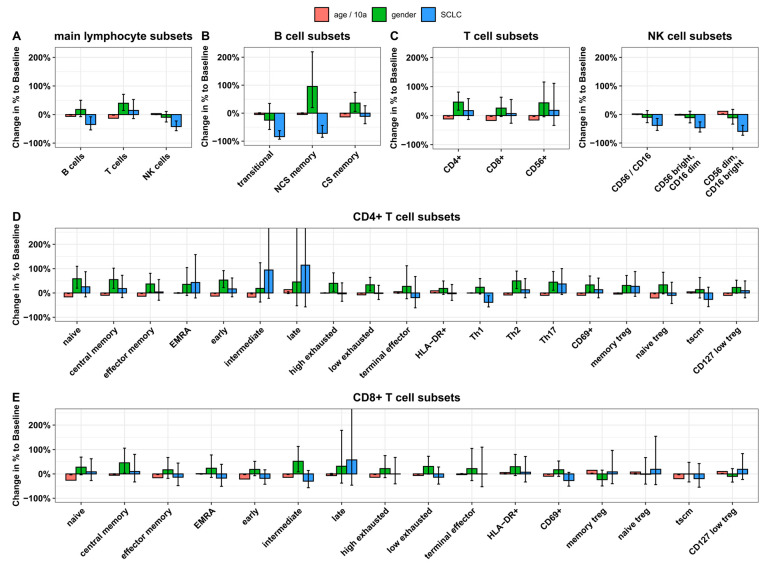
Analysis of lymphocyte subsets with regard to gender, age, and disease (SCLC) in comparison to a healthy control group before the start of immunochemotherapy. The impact of SCLC (blue), as well as gender (green) and age (red), on lymphocyte counts was estimated in multivariable analysis. Levels of SCLC columns indicate percentage deviation from normal values of healthy controls (baseline). Green columns indicate the mean percentage influence of female gender (baseline: male). Levels of age columns reflect percentage deviation per 10 years. Black bars in columns present a 95% confidence interval. Abbreviations: SCLC: small-cell lung cancer; NK: Natural Killer; NCS: non-class switched; CS: class switched; EMRA: effector memory RA+; Th: T helper; Treg: regulatory T; TSCM: stem cell-like memory.

The impact of the disease SCLC, gender, and increasing age per 10-year timeframe on the total count of lymphocytes and their subsets was estimated using a linear regression model on log-transformed cell counts, resulting in multiplicative factors (=coefficient [cB]) in a multivariate model (Figure 1, Supplemental Table S4). 

SCLC had no significant influence on total T lymphocytes (cB: 1.145, *p* = 0.352), but T lymphocytes were elevated by female gender (cB: 1.216, *p* = 0.012) and reduced by increasing age (cB: 0.87, *p* < 0.001). With increasing age, both CD8+ and CD4+ cells had lower values (cB: 0.833, *p* < 0.001 for CD8+ and cB: 0.884, *p* = 0.001 for CD4 + ); however, CD4+ cells were elevated by female gender (cB: 1.465, *p* = 0.001). SCLC had no influence on CD4+ or CD8+ cells. 

Regulatory CD4+ cells (naïve, memory and CD127low) decreased significantly with increasing age but were not influenced by SCLC and gender in multivariable analysis. Regulatory CD8+ T cells showed no significances. 

Further, Th1 cell counts were found with lower levels independently of age and gender in SCLC patients (cB: 0.615, *p* = 0.01). Th2 and Th17 cells were elevated by female gender (cB: 1.498, *p* = 0.001 and cB: 1.445, *p* = 0.007, respectively) and reduced by increasing age (cB: 0.917, *p* = 0.04 and cB: 0.903, *p* = 0.03, respectively). Th17 cells were approximately 40% higher in patients with SCLC in comparison to the healthy control group, but this was not significant (cB: 1.371, *p* = 0.101). Apart from Th1 cells, SCLC did not influence other CD4+ or CD8+ subsets. 

B and NK cells were decreased in patients with SCLC at baseline before the start of ICT compared to healthy controls (cB: 0.653, *p* = 0.016 for B cells, cB: 0.58, *p* < 0.001 for NK cells). This decrease was independent of age and gender. 

Among B-cell subsets, transitional B cells and non-class-switched memory B cells were lower in SCLC patients compared to healthy controls (cB: 0.162, *p* < 0.001 and cB: 0.278, *p* = 0.001, respectively), but non-class-switched B cells were elevated by female gender (cB: 1.956, *p* = 0.008).

Among NK cells, all subsets, CD56+/CD16+, CD56bright/CD16dim, and CD56dim/CD16bright, were significantly lower in patients with SCLC (*p* < 0.005 for each). Lower numbers of CD56dim/CD16bright were additionally dependent on higher age. 

### 2.3. Longitudinal Analysis of Lymphocyte Subsets

**Figure 2:**
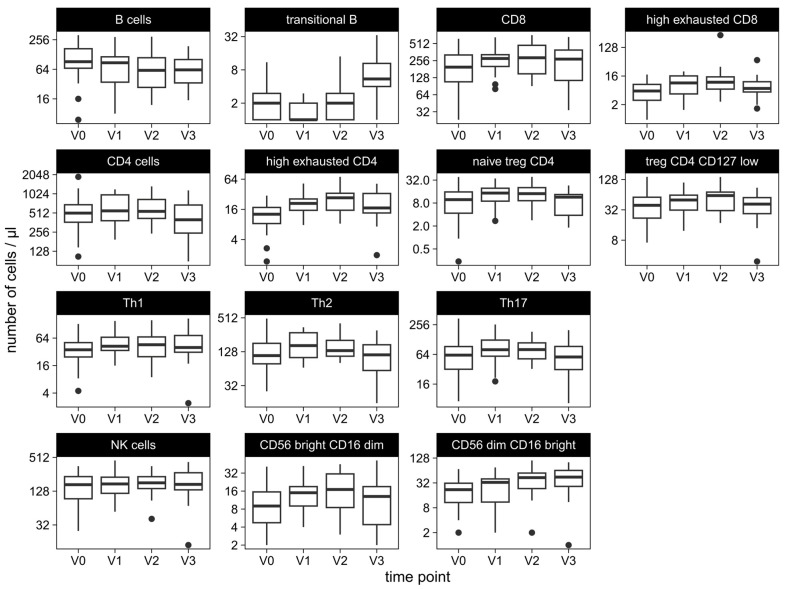
Longitudinal alterations of lymphocyte subsets in course of immunochemotherapy. The distributions of counts of total lymphocytes and subsets measured at the timepoints V0 baseline, (before therapy), V1 after two and at V2 after four cycles of immunochemotherapy, and V3 after two administrations of checkpoint inhibitor maintenance therapy. Abbreviations: Treg: regulatory T; Th: T helper; NK: Natural Killer.

The total number of lymphocytes was 1113 /µL at baseline before start of ICT and did not change significantly during ICT. CD3+ T cells showed a slight but not significant increase from 782 /µL to 1059 /µL (*p* = 0.266), whereas B cells decreased from 91 /µL to 61 /µL after four cycles of ICT (*p* = 0.013) (Table 1, Figure 2).

B cells decreased significantly from baseline (V0) compared to the measurements after four cycles of ICT (V2) (*p* = 0.013) and after two administrations of maintenance therapy (V3) (*p* = 0.02). Among B-cell subsets, we observed significant changes in transitional B cells, non-class-switched memory B cells, and naïve B cells. All numbers of B-cell subsets decreased significantly from baseline (V0) to the measurements after two (V1) and four (V2) cycles of ICT, respectively (*p* < 0.05 for each). After two cycles of maintenance therapy (V3), class-switched and non-class-switched memory B cells decreased further (*p* < 0.001 and *p* = 0.002, respectively), whereas transitional B cells increased again in comparison to the baseline measurement (*p* = 0.002). 

Regarding T cells, overall numbers of CD4+, CD8+, and NK-T cells increased continuously from baseline (V0) to V1 to V2 and decreased from V2 to V3, but these changes were not significant. 

However, high exhausted CD4+ cells and high exhausted CD8+ cells increased significantly after two and four cycles of ICT compared to the baseline measurement (V0) (*p* < 0.001 and *p* = 0.012, respectively, for measurements at V2). After two administrations of maintenance therapy, high exhausted CD4+ (*p* = 0.02) and high exhausted CD8+ T cells (*p* = 0.03) decreased again. No changes were observed in low exhausted CD4+ and CD8+ T cells.

Th cells changed significantly during ICT. Th1, Th2, and Th17 increased continuously but not significantly during ICT (V0 to V2: *p* = 0.055 for Th1, *p* = 0.98 for Th2 and *p* = 0.72 for Th17) and decreased again during maintenance therapy (V2 to V3: *p* = 0.52 for Th1, *p* = 0.09 for Th2 and *p* = 0.03 for Th17). 

Among regulatory CD4+ and CD8+ T cells, naïve, memory, and CD127low subsets were measured. In the course of ICT, regulatory CD8+ subsets did not change and regulatory CD4+ subsets increased slightly but not significantly. Between the measurement after four cycles of ICT (V2) and maintenance therapy (V3), regulatory CD4+/CD127low and naïve regulatory CD4+ T cells decreased again (*p* = 0.021 for regulatory CD4+/CD127low and *p* = 0.015 for naïve regulatory CD4+ T cells). 

The overall number of NK cells was 167 /µL before the start of therapy and did not change significantly during ICT. Among NK subsets, CD56bright/CD16dim and CD56dim/CD16bright cells increased significantly after four cycles of ICT compared to the measurement before the start of ICT (V0 to V2: *p* < 0.001 and *p* = 0.01, respectively). After two cycles of maintenance therapy, CD56bright/CD16dim decreased again (*p* = 0.05), whereas CD56dim/CD16 bright increased further (*p* = 0.02) in comparison to the baseline measurement. 

### 2.4. Predictive Biomarkers for Survival

**Figure 3:**
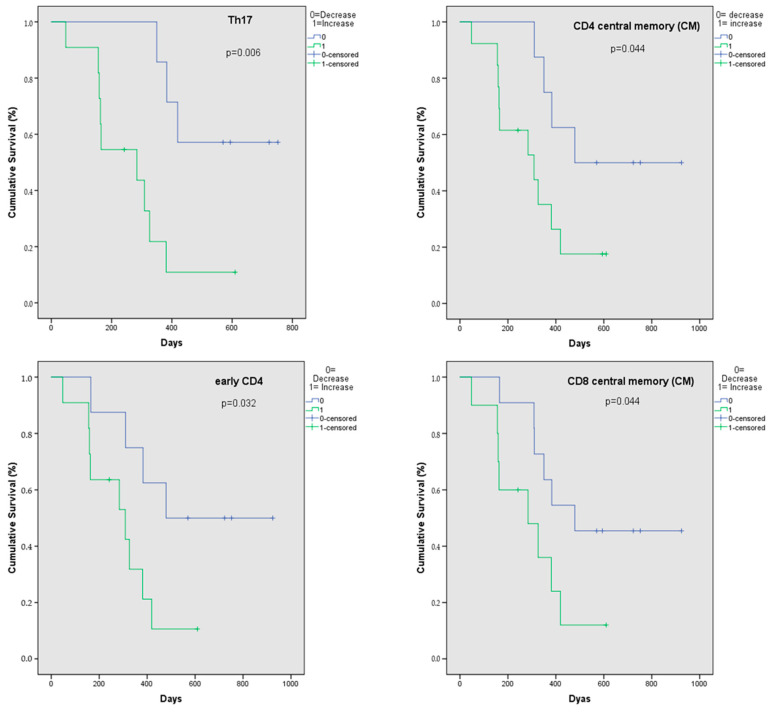
Kaplan–Meier curves for cumulative survival depending on increase (green)/decrease (blue) in T helper (Th) 17, central memory, and early CD4+ cells, as well as central memory CD8+ cells, after two cycles of immunochemotherapy.

Cumulative survival was compared for patients with an increase versus a decrease in lymphocyte subsets. Significant results were observed for Th17, CD4+ central memory, early CD4+, and central memory CD8+ cells (Figure 3). Patients with decreases in these subsets after two cycles of ICT had a longer cumulative survival (*p* = 0.006 for Th17 cells, *p* = 0.044 for CD4+ central memory cells, *p* = 0.032 for early CD4+ cells, and *p* = 0.044 for CD8+ central memory cells, respectively). Median survival for patients with a decrease in Th17cells was not reached, whereas median survival was 284 days for patients with an increase in Th17 cells. Median survival was 479 days for patients with an increase in early CD4+, central memory CD4+, and CD8+ cells, respectively, whereas median survival was 309 days for patients with increases in early and central memory CD4+ cells, respectively, and 284 days for patients with an increase in central memory CD8+ cells. 

## 3. Discussion

In this prospective single-center study, a broad spectrum of lymphocyte subsets during the course of ICT among patients with stage IV SCLC was measured via flow cytometry. The aim of this pilot study was to characterize lymphocyte subsets of SCLC patients in comparison to a healthy cohort. Further, we described changes in lymphocyte subsets during ICT to gain insights into the immune system response under the combination of chemo- and immunotherapy. To find potential biomarkers predicting early immune response to treatment, we correlated decreases/increases in lymphocyte subsets after two cycles of ICT with survival. 

Multivariable analysis showed distinct differences in patients with SCLC in comparison to a healthy control group. Changes were analyzed regarding the influence of disease, age, and gender to overcome possible bias. CD4+ T cells were elevated in women, and decreasing numbers of CD4+ and CD8+ lymphocyte subsets were observed with aging. These findings are in line with studies previously published by our group and others [8,9,10,11]. Further, patients with SCLC had significantly lower numbers of B and NK cells at baseline before the start of ICT, which is consistent with studies concerning patients with other cancers like colorectal carcinoma [9,12,13,14]. Tumor-derived vascular endothelial growth factor (VEGF) may play a role, as this factor not only mediates vascularization but also modulates hematopoiesis [15,16]. It has been reported that patients diagnosed with SCLC have elevated levels of VEGF [17,18]. Although data suggest that VEGF is a promising target, inhibitors of VEGF have limited benefit in SCLC [19,20]. 

A descriptive analysis showed several alterations In the measurement of peripheral blood lymphocytes during ICT. Apart from a significant increase in high exhausted CD4+ and CD8+ T cells, we did not observe significant changes in T lymphocytes and their subsets in the course of ICT. The increase in exhausted CD8+ cells is probably caused by stem cell-like progenitor cells termed “precursor exhausted T cells (T(PEX))” as a result of PD-1-receptor inhibition [21,22,23,24]. This effect is less closely studied for CD4+ T cells but is currently discussed in cancer [25]. Albeit insignificant, a continuous increase in activated T cells like naïve, EMRA, late, and terminal effector CD8+ cells was noticed, reflecting an increasing answer of the immune system. In addition, cells with the late activity marker HLA-DR were also increasing in the course of ICT. Due to the method used, we cannot confirm a specific reaction to ICT; however, no measurements were performed during infection, which might be a confounder. Effector T cells can differentiate to exhausted T cells losing anti-tumor abilities but can be recruited by blocking PD-1 or PD-L1 and restoring their anti-tumor effect. A previous study showed that exhausted T cells were higher in patients not responding to immunotherapy, suggesting that exhausted T cells are a potential prognostic biomarker [26]. Unfortunately, our study cohort was too small to perform a matched-pair analysis between responders and non-responders, though, in Kaplan–Meier analysis, changes in exhausted T cells did not correlate with survival. 

Regulatory T cells are diverse and immunosuppressive and occur in low numbers in peripheral blood [27]. Regulatory T cells can induce immunosuppression through T-lymphocyte-associated protein-4 (CTLA-4), programmed cell death-1 (PD-1), PD-L1, and lymphocyte activation gene 3 (LAG-3), among others [28,29,30]. It is of note that regulatory naïve and CD127low CD4+ T cells increased during ICT and significantly decreased under maintenance with immunotherapy only. We postulate that the increased regulatory immunosuppressive T cells prevent an excessive immune reaction due to the combination of ICT. After the end of chemotherapy and as the tumor becomes smaller, which is accompanied by less inflammation, the number of regulatory T cells decreases again. 

The observed decrease in B cells in patients treated with ICT was previously observed in a course of chemotherapy alone [31]. Subsets such as class-switched and non-class-switched memory B cells are permanently impaired, whereas others like transitional B cells recovered under maintenance immunotherapy in our study cohort. 

NK cells belong to the innate immunity, are powerful effector cells against cancers, and are divided into subsets in which CD56+/CD16+ represent the majority [32,33,34]. In course of ICT NK subsets, CD56bright/CD16dim and CD56dim/CD16bright increased significantly, showing an activation due to ICT. 

In order to change treatment with ICT in case of inefficiency and avoid potential side effects, early response prediction is warranted. Since immune cells differ depending on age and gender, we do not consider the initial baseline values before treatment to be useful predictive biomarkers. Therefore, we correlated increases/decreases in each peripheral blood lymphocyte subset after two cycles of ICT with survival to identify potential early biomarkers in the cellular immune system. According to our results, Th17 cells may have the highest potential to be a good predictive biomarker. Th17 cells are a subset of CD4+ T cells, secrete different cytokines, and play a role in chronic inflammation, autoimmune diseases like Multiple Sclerosis, and cancer [35,36]. Concerning cancer, there are data showing that Th17 cells can promote tumorigenesis and progression depending on the tumor entity [37,38,39]. Th17 cells can contribute to angiogenesis and tumor vascularization [40]. In NSCLC, high levels of Th17 and interleukin (Il)-17 have been suggested as prognostic markers as high levels are associated with advanced stages [41,42,43,44]. Apart from lung cancer, an increase in Th17 cells was also described in other tumor entities like cervical carcinoma. Theobald et al. reported that chemoradiotherapy increased Th17 cells in the peripheral blood of cervical cancer patients and that higher Th17 cells after therapy were associated with recurrent cervical cancer [39]. 

In our study, a decrease in Th 17 cells after two cycles of ICT was associated with a significantly better cumulative survival. In multivariable analysis, the number of Th17 cells was not significant but still approximately 40% higher in patients with SCLC compared to the healthy control cohort. An explanation for the beneficial effect of Th17 decrease might be that these cells normalize to the values found in the healthy control cohort, and this correlates with a good response to therapy. Further, as previously demonstrated [45], assuming a positive correlation between Th17 cells in peripheral blood and in tumor tissue, Th17 cells might also decrease in the tissue. However, this cannot be proven by our study, as we did not perform comparative analysis of peripheral blood and histological specimens. With the decrease, the potentially tumor-protective effect of Th17 cells also disappears, and the tumor cells become more vulnerable to attack by the immune system. Therefore, we predict that the dynamic under ICT correlates with response to therapy.

Limitations of our study are its single-center character and relatively small number of patients. However, we performed the same tests in a homogenous study cohort, all of whom were presenting with stage IV SCLC and receiving the same therapy. A matched-pair analysis with patients responding and not responding to ICT could not be performed due to the low number of patients. Further, analysis was only performed in peripheral blood but not in tumor tissue, so we cannot conclude on local immune response in the tumor. As mentioned above, several studies, especially on immunomodulatory T cells in NSCLC, exist, but there are only a few on SCLC and the analysis of changes in lymphocyte subsets during the course of therapy. 

In conclusion, the monitoring of lymphocyte subsets throughout ICT in patients with SCLC and in comparison to a healthy control group revealed notable alterations in various lymphocyte subsets. Specifically, a decrease in Th17 cells after two cycles of ICT exhibited an association with a significantly prolonged survival. Based on our findings in this pilot study, we suggest that Th17 cells are a good candidate for further investigation as a potential predictive biomarker for treatment response in patients receiving ICT for lung cancer. 

## 4. Materials and Methods

### 4.1. Study Population

In total, 32 adult patients with stage IV SCLC diagnosed at University Hospital Augsburg between 2020 and 2022 were included. Patients with a history of autoimmune disease, any immunodeficiency disorder, or under immunosuppressive therapy were excluded. All included patients were treated with ICT with carboplatin d1/etoposide d1-3 in combination with atezolizumab d1. The standard treatment protocol comprises four cycles of ICT, followed by maintenance therapy with atezolizumab mono every three weeks. During the three days of chemotherapy, patients received antiemetic prophylaxis with 8 mg of dexamethasone limited to these three days. A total of 75 healthy adult blood donors from the blood bank at University Hospital Augsburg served as a control group. This study was conducted according to the guidelines of the Declaration of Helsinki and approved by the Institutional Review Board of the University Hospital Augsburg, Germany (protocol code 2020-10, approved 25 April 2020). Informed consent was obtained from all patients and healthy control participants involved in this study.

### 4.2. Analysis of Lymphocytes and Subsets

Peripheral blood (EDTA) was drawn at four designated time points signed V0 to V3, before start of ICT (baseline, V0), after two (V1) and after four (V2) cycles of ICT, and after two administrations of maintenance therapy with atezolizumab only (V3). In some cases, measurements were not performed at all time points due to early death or other reasons, e.g., progressive disease, infection (defined by suspected infect focus with or without fever and the indication for treatment with antibiotics) or delivery failures due to the COVID-19 pandemic (see Supplemental Figure S1 for details). At baseline (V0), lymphocytes were measured in all 32 patients; at time point V1, they were measured in 21 patients; at time point V2, they were measured in 18 patients; and at time point, they were measured V3 in 15 patients. Blood samples were processed within 24 h and prepared for flow cytometry using a Navios EX Flow Cytometer (Beckman Coulter, Brea, CA, USA). The gating strategy was applied as previously published by our group [8,9]. Antibodies derived from Beckman Coulter and BioLegend (San Diego, CA, USA) were used for cell staining. Total lymphocyte cell counts were measured using Stem-Count beads (Stem-Kit Reagents, Beckman Coulter). 

We used flow cytometry to determine the absolute numbers of B (CD19+) and T (CD3+) cells and Natural Killer (NK) (CD56+) and NK-like T (CD3+ and CD56+) cells and their respective subsets. For further details on lymphocyte subset-specific immunophenotypes and fluorochrome–antibody conjugates, see Supplemental Tables S1 and S2. In brief, B lymphocytes were divided into naïve, class-switched memory; non-class-switched memory; and transitional B cells. CD4+ and CD8+ T cells were subdivided into naïve, central memory (CM); effector memory (EM); effector memory RA (EMRA); early, intermediate, late, and terminal effector (TE); high and low exhausted; HLA-DR+; CD69+; and stem cell-like memory (TSCM) T cells. Among CD4+ cells, T helper (Th) cells 1, 2, and 17 were measured. Further, naïve, memory and CD127low regulatory CD4+ or CD8+ T cells were analyzed. NK lymphocytes were subdivided into three functional groups (CD56+ CD16+, CD56dim/CD16bright, and CD56bright/CD16dim). 

### 4.3. Statistical Analysis

Statistical analyses were performed with SPSS 24.0 (SPSS Inc., Chicago, IL, USA) and R 4.3.2 (R Foundation for Statistical Computing, Vienna, Austria). The results of descriptive analysis are given as median and interquartile ranges. 

Friedman’s test was used to detect significant changes in lymphocyte subsets in a longitudinal comparison. In 15 patients, lymphocyte subsets were measured at all four time points. All other patient´s measurements were excluded from Friedman’s test. Significantly changing subsets, as well as main lymphocyte subsets, T helper cells and regulatory T cells, were further analyzed via a Wilcoxon test for associated samples. We compared the results after two (V1), four (V2), and six (V3) cycles of therapy, respectively, to the ones at baseline before start of treatment (V0) and among each other. For the comparison between time points, all available measurements were used. *p*-values < 0.05 were regarded as statistically significant. 

A linear regression model on log-transformed cell counts was used to analyze the independent influence of disease (SCLC), age, and gender in patients with SCLC before the start of ICT compared to the healthy control cohort (not matched in age and gender). 

For Kaplan–Meier curves, the number of lymphocyte subsets at baseline (V0, before the start of treatment) was divided by the number at V1 (after two cycles of ICT). A ratio higher than 1 means an increase, a ratio smaller than 1 means a decrease. Cumulative survival was compared for patients with an increase versus a decrease in lymphocyte subsets. 

## Figures and Tables

**Table 1:** Absolute numbers of lymphocyte subsets in SCLC patients in the course of immunochemotherapy.

Variable	V0 Baseline Median (IQR)	V1 after Two Cycles ICT Median (IQR)	V2 after Four Cycles ICT Median (IQR)	V3 after Two Cycles CPI Maintenance Median (IQR)
**Total lymphocytes**	1113.5 (866.25–1379)	1256 (854–1638)	1264 (941.5–1638.5)	1102.5 (681–1507.25)
**Total T cells (CD3+)**	782 (575.5–1008.25)	923 (708–1136)	1059 (702.5–1249.5)	809.5 (519.25–1137.25)
**Cytotoxic T cells** **(CD8+)**	196 (108–322)	279 (202–326)	287 (149–477)	276 (115–394)
**CD8+ naive**	56.6 (45.2–104.3)	95 (71.1–134.2)	110.9 (51.8–123.6)	72.8 (28–127.1)
**CD8+ CM**	22.5 (15.7–41.2)	32.1 (18.4–59.2)	25.3 (15.8–52.1)	20.6 (14.6–40.6)
**CD8+ EM**	17.2 (10–28.6)	26.3 (19–40.5)	24.6 (11.9–52.7)	17 (12.7–45.5)
**CD8+ EMRA**	53.2 (22.9–107.4)	55.5 (41.6–99.5)	88.1 (38–239.6)	97.5 (20.5–171.8)
**CD8+ TE**	19.8 (5.2–70.1)	29.9 (9.9–94.6)	40.5 (10–178.6)	57.7 (4.9–109.2)
**CD8+ TSCM**	1.3 (0.5–2.4)	1.6 (1–2.3)	1 (0.4–2.3)	0.9 (0.7–2.5)
**CD8+ early**	77.5 (55.9–114.9)	121.6 (94.7–173.4)	119.3 (69.6–140.8)	83.2 (37.1–127.1)
**CD8+ intermediate**	8.4 (3.3–13.4)	9.6 (7.6–14.8)	12.3 (7–20.1)	10.2 (5–20.8)
**CD8+ late**	37 (4.4–121.6)	69 (20.7–172.7)	133.9 (21–257.2)	146.7 (15.5–243.2)
**CD8+ high exhausted**	5.4 (2.7–8.7)	9.7 (4.3–16.1)	10.2 (6.2–15.1)	6.5 (5–10.6)
**CD8+ low exhausted**	66.6 (37–116.1)	84.1 (54.3–10.5.4)	128.3 (40.8–181.4)	68.9 (42.4–155.9)
**CD8+ HLA-DR**	74.1 (36.9–182.3)	98.1 (52.7–209.9)	108.7 (65.6–313.6)	127.2. (43.7–252.7)
**CD69+ CD8+**	36 (21–59.8)	35.9 (28.1–57)	42.5 (30.9–74)	33.2 (26.3–47.4)
**Naive Treg CD8+**	0.3 (0.1–0.4)	0.4 (0.3–0.8)	0.3 (0.2–0.4)	0.2 (0.2–0.5)
**Memory Treg CD8+**	0.7 (0.3–1.1)	0.9 (0.4–1.1)	0.8 (0.5–1.4)	0.5 (0.4–0.9)
**Treg CD8+** **CD127low**	0.9 (0.6–1.6)	1.2 (0.8–2)	1.1 (0.7–1.7)	0.8 (0.4–1.5)
**T helper cells (CD4+)**	509.5 (365.9–689.3)	552 (386–986)	542 (421–834)	404 (247–682)
**CD4+ naive**	206.5 (147–290)	278.2 (179.4–539)	273.5 (178.7–382.8)	149.8 (95.2–292.2)
**CD4+ CM**	175 (104.5–251.5)	202.8 (141.5–294.4)	189.5 (140.5–293.1)	161.7 (114.8–284.8)
**CD4+ EM**	60.7 (42.2–93)	70.8 (44.9–90.6)	69.4 (35.4–97.4)	56.5 (47.5–80.4)
**CD4+ EMRA**	7.9 (4.4–15.7)	9.4 (5.5–27.7)	10.8 (5.1–25.2)	9.2 (4.3–16.9)
**CD4+ TE**	3.5 (1.1–9.7)	5.8 (2.7–12.4)	5.7 (3.2–9.4)	3.9 (1.5–6.7)
**CD4+ TSCM**	1.3 (1–2.2)	1.9 (0.9–3.6)	0.9 (0.5–1.4)	0.8 (0.5–1.9)
**CD4+ early**	439.8 (274.3–638.2)	566.2 (351.8–936.6)	465.3 (362.6–692.9)	370 (204.5–605.9)
**CD4+ intermediate**	0.1 (0.1–0.4)	0.2 (0.1–0.6)	0.1 (0–0.3)	0.1 (0–0.6)
**CD4 late**	1.4 (0.2–17.5)	7.3 (0.6–35.6)	5.4 (0.3–27.9)	7.1 (1.7–30.3)
**CD4+ high exhausted**	12.8 (8.4–17.5)	21 (15.3–25.8)	27.2 (15.4–33.5)	17.1 (13.6–33.2)
**CD4+ low exhausted**	112.6 (68.9–171.8)	120.7 (93.6–178.7)	144.9–77-182.2)	143.9–82.6–182.2)
**CD4+ HLA-DR**	58.2 (27.2–111.6)	61.9 (37.6–10.9.9)	76.8 (40.5–116.2)	65.7 (45–95.6)
**Naive Treg CD4+**	9.8 (4.3–15.6)	14.6 (9–19.6)	14.1 (9.4–20.3)	11.4 (3.8–13.4)
**Memory Treg CD4+**	25.1 (17.1–40.8)	32.6 (22.1–42.1)	37.7 (22.8–49.7)	30.7 (21.4–43.4)
**Treg CD4+ CD127low**	39.4 (21.9–57)	50.2 (31.6–62.7)	61.5 (30.9–72.4)	41.6 (27–56.2)
**CD69+ CD4+**	61.7 (40.4–79.9)	56.6 (46.4–79.1)	58.6 (40.5–66.9)	42.5 (20.9–75.2)
**CD4+ CD8+**	6.7 (3.2–8.4)	6.9 (3.3–10.7)	9.9 (4.1–10.7)	5.7 (2.4–7.5)
**Th 1**	35.5 (24.7–51)	42.5 (34.3–66.9)	45.8 (25–68.1)	39.8 (31.4–73.2)
**Th 2**	109 (78–180.3)	164.3 (100.8–278)	134 (106.5–203.7)	112.7 (59.4–172.2)
**Th 17**	61.9 (32–92.4)	79.5 (58.8–124.8)	80.4 (52–109.9)	56.9 (31.9–92.7)
**NK like T cells**	15 (8.5–44.5)	32 (16–79)	35 (14.5–84)	25.5 (12.5–74.75)
**Total B cells (CD19+)**	91.5 (68–168.25)	87 (35–115)	61 (27.5–111)	62.5 (34–101.25)
**Naïve B cells**	64.5 (40–105)	56 (18–81)	41 (18–81.5)	45.5 (26.5–72-5)
**Non-class-switched memory** **B cells**	5 (2–13.5)	4 (2–7)	2 (1.5–4)	1 (1–4.8)
**Class-switched** **memory B cells**	15 (7.8–24)	12 (7–22)	11 (5–22)	5 (2.8–13.8)
**Transitional B** **cells**	1 (1–3)	1 (0–1)	0 (0–1.5)	5.5 (4–10.5)
**Natural killer cells**	167 (93–232)	172 (117–227)	180 (142–232)	169 (135–272)
**CD56+ CD16+**	127.5 (59.5–194)	109 (76–170)	114 (91–148.5)	104 (74.3–174.8)
**CD56bright CD16dim**	9 (4.8–15.5)	15 (9–19)	17 (8.5–31)	13 (4.5–19)
**CD56dim CD16bright**	22 (10.8–31.5)	33 (11–40)	43 (23.5–55.5)	44.5 (26.5–64-3)

Cell counts are given as median value/µL. V0: baseline (measurement before start of immunochemotherapy); V1: after two cycles; V2: after four cycles; V3: after two administrations of checkpoint inhibitor maintenance therapy. Abbreviations: SCLC: small-cell lung cancer; IQR: interquartile range; CM: central memory; EM: effector memory; EMRA: effector memory RA+; TE: terminal effector; TSCM: stem cell-like memory T; Treg, regulatory T; Th: T helper; NK: Natural Killer.

## Data Availability

The data presented in this study are available on request from the corresponding author due to legal reasons.

## Supplementary Materials

The following supporting information can be downloaded at: https://www.mdpi.com/article/10.3390/ijms25105056/s1.
