# Supplementary material for: Alterations in Peripheral Lymphocyte Subsets under Immunochemotherapy in Stage IV SCLC Patients: Th17 Cells as Potential Early Predictive Biomarker for Response
Source: Int J Mol Sci. 2024 May 7;25(10):5056. doi: 10.3390/ijms25105056 (PMC11121216; doi:10.3390/ijms25105056)
Supplement: Supplementary file 1 [file ijms-25-05056-s001.zip › ijms-2966703-supplementary.pdf]

Supplemental Figure S1. Flowchart of therapy cycles and drop out

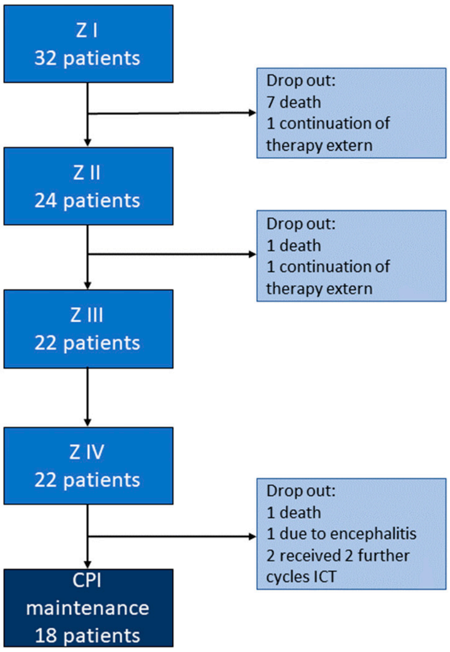

Figure legend: Patients were treated with immunochemotherapy (ICT) cycles (Z) I to IV (carboplatin, etoposide and atezolizimab), followed by maintenance with checkpoint inhibitor (CPI) atezolizumab mono. One patient developed encephalitis as a side effect of CPI.

Supplemental Table S1. Identification and gating strategy for detection of main lymphocyte types and their subsets using flow cytometry.

Lymphocytes	Immunophenotype
**Total B lymphocytes (CD19+)**	**CD3- CD19+**
Naïve B cells	IgD+ CD27-
Transitional B cells	CD24+ CD38+
Non-class switched memory B cells	IgD+ CD27+
Class switched memory B cells	IgD- CD27+
**Total T lymphocytes (CD3+)**	**CD3+ CD19-**
**Natural killer-like T (NKT) cells (CD56+ T cells)**	**CD3+ CD56+**
**T helper lymphocytes (CD4+)**	**CD3+ CD4+**
Naive	CD62L+ CD45RA+
Central memory	CD62L+ CD45RA-
Effector memory	CD62L- CD45RA-
Effector memory RA+ (EMRA)	CD62L- CD45RA+
Early activated	CD28+ CD27+
Intermediate activated	CD28- CD27+

Late activated	CD28- CD27-
High exhausted	CD279++ / CD279bright
Low exhausted	CD279+ / CD279dim
Terminal effector	CD279- CD57+
HLA DR+ (activated)	HLA-DR+
CD69+ (activated)	CD69+
Th1	CXCR3+ CCR4- CCR5+ CCR6-
Th2	CXCR3- CCR4+ CCR5- CCR6-
Th17	CXCR3- CCR4+ CCR5- CCR6+
CD127 low regulatory	CD25+ CD127 low
Naive regulatory	CD45RA-
memory reulatory	CD45RA+
stem cell-like memory (tscm)	CD27+ CD28+ CD45RA+vCD57- CD62L+ CD95+
**Cytotoxic lymphocytes (CD8+)**	**CD3+ CD8+**
Naive	CD62L+ CD45RA+
Central memory	CD62L+ CD45RA-
Effector memory	CD62L- CD45RA-
Effector memory RA+ (EMRA)	CD62L- CD45RA+
Early activated	CD28+ CD27+
Intermediate activated	CD28- CD27+
Late activated	CD28- CD27-
High exhausted	CD279++ / CD279bright
Low exhausted	CD279+ / CD279dim
Terminal effector	CD279- CD57+
HLA-DR+ (activated)	HLA-DR+
CD69+ (activated)	CD69+
CD127 low regulatory	CD25+ CD127 low
Naive regulatory	CD45RA-
memory reulatory	CD45RA+
stem cell-like memory (tscm)	CD27+ CD28+ CD45RA+vCD57- CD62L+ CD95+
**Natural killer lymphocytes (NK)**	**CD56+ CD3-**
CD56+/CD16+	CD56+/CD16+
CD56bright/CD16dim	CD56bright/CD16dim
CD56dim/CD16bright	CD56dim/CD16bright

Supplemental Table S2. Information on antigens and fluorochrome-antibody conjugates

	Antigen	Fluorochrome	Clone	Isotype	Article no. Beckmann Coulter/ Biolegend
**Tube 01**	IgD	FITC	IA6-2	Mouse IgG2a	B30652
	CD27	PE	1A4CD27	Mouse IgG1	B96790
	CD16	ECD	3G8	Mouse IgG1	B49216
	CD56	PC5.5	N901	Mouse IgG1	B49189
	CD38	PC7	LS198-4-3	Mouse IgG1	B49198
	CD24	APC	ALB9	Mouse IgG1	A87785
	CD3	APC-AF750	UCHT1	Mouse IgG1	A94680
	CD19	Pacific Blue	J3-119	Mouse IgG1	B49213
	CD45	Krome Orange	J33	Mouse IgG1	B36294
**Tube 02**	CD45RA	FITC	ALB11	Mouse IgG1	A07786
	CD95	PE	UB2	Mouse IgG1	IM1739
	CD62L	ECD	DREG56	Mouse IgG1	IM2713U
	CD279	PC5.5	PD1.3	Mouse IgG2b	B36123
	CD28	PC7	CD28.2	Mouse IgG1	B23313
	CD27	APC	1A4CD27	Mouse IgG1	B09983
	CD8	APC-AF700	B9.11	Mouse IgG1	B49181
	CD3	APC-AF750	UCHT1	Mouse IgG1	A94680
	CD57	Pacific Blue	NC1	Mouse IgM	A74779
	CD4	Krome Orange	13B8.2	Mouse IgG1	A96417
**Tube 03**	CD45RA	FITC	ALB11	Mouse IgG1	A07786
	CD25	PE	B1.49.9	Mouse IgG2a	A07774
	CD69	ECD	TP1.55.3	Mouse IgG2b	6607110
	CD127	APC	R34.34	Mouse IgG1	B42026
	CD8	APC-AF700	B9.11	Mouse IgG1	B49181
	HLA-DR	Pacific Blue	Immu-357	IgG1	B36291
	CD4	Krome Orange	13B8.2	Mouse IgG1	A96417
**Tube 04**	CD183 (CXCR3)	AF488	G025H7	Mouse IgG1	B68144
	CD195 (CCR5)	PE	J418F1	Rat IgG2b	359106
	CD196 (CCR6)	PC7	B-R35	Mouse IgG2a	B68132
	CD3	APC-AF750	UCHT1	Mouse IgG1	A94680
	CD194 (CCR4)	BV421	L291H4	Mouse IgG1 kappa	359414
	CD4	Krome Orange	13B8.2	Mouse IgG1	A96417

Supplemental Table S3. Patient characteristics

Patient	Sex	Age at diagnosis	Baseline CRP (Ref.: 0-0.5 mg/dl)	Brain metastases at diagnosis	Distant metastases other than brain at diagnosis	Irradiation during treatment with ICT or CPI maintenance
1	m	67	1.2	no	LYMPH, PUL	Prophylactic whole brain
2	w	79	2	no	OSS	Prophylactic whole brain
3	w	66	normal	no	HEP, ADR, PUL	no
4	w	80	normal	no	PLE, LYMPH, OSS, HEP	no
5	w	81	normal	no	LYMPH, PLE	no
6	m	80	3.4	Yes (1)	LYMPH, PLE, ADR, OSS	Irradiation of brain and bone metastases
7	w	60	normal	no	PUL, LYMPH	Prophylactic whole brain
8	w	50	normal	no	HEP, ADR	no
9	m	70	normal	no	PLE, LYMPH	no
10	m	72	11.5	no	PUL, LYMPH, HEP, PLE, OSS, OTH	Irradiation of primary lung tumor
11	m	76	normal	no	LYMPH, PLE	Prophylactic whole brain
12	w	44	2.2	no	PLE, LYMPH	Prophylactic whole brain
13	w	64	1.4	Yes (3)	PUL	Radiation of brain metastases
14	m	64	normal	no	LYMPH, HEP, OSS	no
15	w	71	normal	no	LYMPH, PUL, OSS	no
16	m	78	7.5	no	LYMPH, HEP, OTH	no
17	m	74	1.4	no	LYMPH, HEP	Prophylactic whole brain
18	m	64	normal	no	PUL, OSS	no
19	m	79	normal	no	PLE, LYMPH	no
20	w	71	normal	Yes (multiple)	PLE	Whole brain radiation
21	m	70	2.2	no	LYMPH, PLE, OSS	no
22	w	83	normal	no	PUL, PLE, LYMPH	no
23	m	74	17.9	no	LYMPH, ADR, PER, OTH	no
24	m	69	1.6	no	PUL, LYMPH, HEP, OSS	no
25	m	62	9.7	no	PUL, HEP, ADR, OTH	no
26	m	69	normal	no	PUL, LYMPH, HEP	no
27	m	67	normal	Yes (3)	PUL, ADR	no

28	m	67	normal	no	PUL, LYMPH, HEP	no
29	w	64	normal	no	PUL, LYMPH	no
30	m	82	11.4	no	PUL, HEP, OSS	Irradiatio of bone metastasis
31	m	59	2.0	Yes (3)	PUL, LYMPH, HEP, OSS, OTH	Irradiation of brain and bone metastases
32	w	69	1.3	no	LYMPH, PLE, PUL, OTH	no

Supplemental Table S4. Impact of gender, small cell lung cancer (SCLC), and increasing age (per 10 years) on total count of lymphocytes and their subsets estimated as multiplicative factor (= coefficient B) in a multivariable model.

	Variables	Coefficients B (95% CI)	p
**Total Lymphocytes**	gender	1.216 (1.045-1.415)	0.012
	Age (/10 years)	0.914 (0.986-0.996)	0.001
	SCLC	0.930 (0.748-1.154)	0.505
**Total T cells (CD3+)**	gender	1.397 (1.143-1.708)	0.001
	Age (/10 years)	0.870 (0.979-0.993)	< 0.001
	SCLC	1.145 (0.859-1.526)	0.352
**Cytotoxic T cells (CD8+)**	gender	1.258 (0.969-1.634)	0.084
	Age (/10 years)	0.833 (0.973-0.991)	< 0.001
	SCLC	1.069 (0.736-1.553)	0.723
**CD8+ naive**	gender	1.277 (0.964-1.691)	0.088
	Age (/10 years)	0.741 (0.961-0.980)	< 0.001
	SCLC	1.086 (0.727-1.623)	0.685
**CD8+ CM**	gender	1.456 (1.030-2.059)	0.034
	Age (/10 years)	0.944 (0.982-1.006)	0.346
	SCLC	1.099 (0.670-1.804)	0.706
**CD8+ EM**	gender	1.170 (0.817-1.675)	0.388
	Age (/10 years)	0.843 (0.971-0.996)	0.008
	SCLC	0.865 (0.518-1.446)	0.577
**CD8+ EMRA**	gender	1.234 (0.856-1.779)	0.257
	Age (/10 years)	1.011 (0.988-1.014)	0.871
	SCLC	0.827 (0.490-1.395)	0.472
**CD8+ TE**	gender	1.217 (0.723-2.049)	0.456
	Age (/10 years)	0.970 (0.979-1.015)	0.738
	SCLC	0.997 (0.473-2.100)	0.994

**CD8+ TSCM**	gender	0.994 (0.669-1.477)	0.976
	Age (/10 years)	0.804 (0.965-0.992)	0.002
	SCLC	0.805 (0.454-1.425)	0.452
**CD8+ early**	gender	1.185 (0.926-1.516)	0.176
	Age (/10 years)	0.790 (0.968-0.985)	< 0.001
	SCLC	0.820 (0.575-1.169)	0.270
**CD8+ intermediate**	gender	1.519 (1.083-2.130)	0.016
	Age (/10 years)	0.859 (0.973-0.997)	0.012
	SCLC	0.702 (0.432-1.142)	0.152
**CD8+ late**	gender	1.316 (0.622-2.782)	0.469
	Age (/10 years)	0.931 (0.967-1.019)	0.588
	SCLC	1.576 (0.537-4.623)	0.404
**CD8+ high exhausted**	gender	1.218 (0.847-1.753)	0.285
	Age (/10 years)	0.862 (0.973-0.998)	0.023
	SCLC	0.997 (0.592-1.677)	0.990
**CD8+ low exhausted**	gender	1.308 (0.992-1.725)	0.057
	Age (/10 years)	0.935 (0.984-1.003)	0.175
	SCLC	0.864 (0.581-1.284)	0.466
**CD8+ HLA-DR**	gender	1.297 (0.933-1.802)	0.120
	Age (/10 years)	1.056 (0.994-1.017)	0.349
	SCLC	1.071 (0.668-1.717)	0.773
**CD69+ CD8+**	gender	1.172 (0.899-1.530)	0.238
	Age (/10 years)	0.904 (0.981-0.999)	0.034
	SCLC	0.727 (0.497-1.065)	0.101
**Naive Treg CD8+**	gender	0.985 (0.581-1.671)	0.955
	Age (/10 years)	1.078 (0.989-1.026)	0.421
	SCLC	1.190 (0.558-2.538)	0.651
**Memory Treg CD8+**	gender	0.763 (0.504-1.155)	0.199
	Age (/10 years)	1.148 (0.999-1.029)	0.061
	SCLC	1.084 (0.598-1.966)	0.788
**Treg CD8+ CD127 low**	gender	0.899 (0.664-1.217)	0.488
	Age (/10 years)	1.099 (0.999-1.020)	0.079
	SCLC	1.189 (0.770-1.835)	0.432
**T helper cells (CD4+)**	gender	1.465 (1.186-1.810)	0.001
	Age (/10 years)	0.884 (0.980-0.995)	0.001
	SCLC	1.172 (0.866-1.585)	0.302
**CD4+ naive**	gender	1.584 (1.197-2.097)	0.002
	Age (/10 years)	0.840 (0.973-0.992)	0.001
	SCLC	1.254 (0.840-1.873)	0.264
**CD4+ CM**	gender	1.549 (1.189-2.019)	0.001
	Age (/10 years)	0.905 (0.981-0.999)	0.035
	SCLC	1.182 (0.809-1.727)	0.383

**CD4+ EM**	gender	1.370 (1.037-1.809)	0.027
	Age (/10 years)	0.866 (0.976-0.995)	0.004
	SCLC	1.042 (0.700-1.550)	0.839
**CD4+ EMRA**	gender	1.354 (0.897-2.044)	0.148
	Age (/10 years)	0.992 (0.985-1.014)	0.913
	SCLC	1.431 (0.794-2.579)	0.231
**CD4+ TE**	gender	1.271 (0.763-2.118)	0.354
	Age (/10 years)	1.051 (0.987-1.023)	0.585
	SCLC	0.808 (0.389-1.676)	0.563
**CD4+ TSCM**	gender	1.139 (0.795-1.632)	0.474
	Age (/10 years)	1.046 (0.992-1.017)	0.478
	SCLC	0.736 (0.438-1.237)	0.245
**CD4+ early**	gender	1.530 (1.220-1.919)	< 0.001
	Age (/10 years)	0.871 (0.979-0.994)	0.001
	SCLC	1.167 (0.843-1.616)	0.349
**CD4+ intermediate**	gender	1.187 (0.628-2.242)	0.594
	Age (/10 years)	0.829 (0.960-1.003)	0.096
	SCLC	1.944 (0.779-4.849)	0.152
**CD4 late**	gender	1.452 (0.474-4.450)	0.510
	Age (/10 years)	1.136 (0.974-1.053)	0.520
	SCLC	2.143 (0.429-10.712)	0.350
**CD4+ high exhausted**	gender	1.397 (1.069-1.827)	0.015
	Age (/10 years)	0.992 (0.990-1.009)	0.871
	SCLC	0.965 (0.658-1.415)	0.854
**CD4+ low exhausted**	gender	1.336 (1.087-1.641)	0.006
	Age (/10 years)	0.923 (0.985-0.999)	0.030
	SCLC	0.979 (0.730-1.315)	0.888
**CD4+ HLA-DR**	gender	1.185 (0.941-1.493)	0.148
	Age (/10 years)	1.089 (1.000-1.017)	0.039
	SCLC	0.968 (0.695-1.348)	0.846
**Naive Treg CD4+**	gender	1.334 (0.963-1.850)	0.083
	Age (/10 years)	0.794 (0.966-0.988)	< 0.001
	SCLC	0.902 (0.565-1.441)	0.663
**Memory Treg CD4+**	gender	1.334 (0.963-1.850)	0.083
	Age (/10 years)	0.794 (0.966-0.988)	< 0.001
	SCLC	0.902 (0.565-1.441)	0.663
**Treg CD4+ CD127 low**	gender	1.227 (0.987-1.525)	0.065
	Age (/10 years)	0.901 (0.982-0.997)	0.008
	SCLC	1.094 (0.801-1.495)	0.567
**CD69+ CD4+**	gender	1.331 (1.043-1.698)	0.022
	Age (/10 years)	0.905 (0.982-0.999)	0.022
	SCLC	1.135 (0.800-1.611)	0.474

**CD4+ CD8+**	gender	1.546 (1.057-2.263)	0.025
	Age (/10 years)	1.039 (0.991-1.017)	0.569
	SCLC	1.316 (0.764-2.268)	0.320
**Th 1**	gender	1.235 (0.958-1.592)	0.102
	Age (/10 years)	0.996 (0.991-1.008)	0.925
	SCLC	0.615 (0.427-0.886)	0.010
**Th 2**	gender	1.498 (1.181-1.899)	0.001
	Age (/10 years)	0.917 (0.983-1.000)	0.040
	SCLC	1.131 (0.803-1.591)	0.478
**Th 17**	gender	1.445 (1.111-1.880)	0.007
	Age (/10 years)	0.903 (0.981-0.999)	0.030
	SCLC	1.371 (0.939-2.002)	0.101
**NK like T cells**	gender	1.439 (0.960-2.158)	0.077
	Age (/10 years)	0.848 (0.970-0.998)	0.023
	SCLC	1.181 (0.660-2.112)	0.572
**Total B cells (CD19+)**	gender	1.178 (0.926-1.500)	0.180
	Age (/10 years)	0.936 (0.985-1.002)	0.123
	SCLC	0.653 (0.462-0.921)	0.016
**Naïve B cells**	gender	1.174 (0.832-1.655)	0.357
	Age (/10 years)	0.958 (0.984-1.008)	0.483
	SCLC	0.616 (0.377-1.006)	0.053
**Non-class switched memory B cells**	gender	1.956 (1.198-3.193)	0.008
	Age (/10 years)	0.954 (0.978-1.012)	0.584
	SCLC	0.278 (0.138-0.561)	0.001
**Class switched memory B cells**	gender	1.361 (1.062-1.744)	0.016
	Age (/10 years)	0.867 (0.977-0.994)	0.002
	SCLC	0.887 (0.622-1.265)	0.505
**Transitional B cells**	gender	0.750 (0.417-1.348)	0.332
	Age (/10 years)	0.953 (0.975-1.016)	0.643
	SCLC	0.162 (0.070-0.375)	< 0.001
**Natural killer cells**	gender	0.905 (0.740-1.107)	0.328
	Age (/10 years)	1.033 (0.996-1.010)	0.359
	SCLC	0.580 (0.435-0.774)	< 0.001
**CD56+ CD16+**	gender	0.899 (0.712-1.136)	0.370
	Age (/10 years)	1.020 (0.994-1.010)	0.635
	SCLC	0.616 (0.441-0.860)	0.005
**CD56bright/CD16dim**	gender	0.887 (0.705-1.115)	0.301
	Age (/10 years)	0.976 (0.990-1.006)	0.546
	SCLC	0.533 (0.384-0.739)	< 0.001
**CD56dim/CD16bright**	gender	0.882 (0.661-1.177)	0.391
	Age (/10 years)	1.110 (1.000-1.021)	0.043
	SCLC	0.407 (0.270-0.615)	< 0.001

Abbreviations: CI: confidence interval, CM: central memory, EM: effector memory, EMRA: effector memory RA+, TE: terminal effector, TSCM: stem cell-like memory T, Treg, regulatory T, Th: T helper, NK: Natural Killer
